# Changes in the equine facial repertoire during different orthopedic pain intensities

**DOI:** 10.1038/s41598-023-50383-y

**Published:** 2024-01-02

**Authors:** Katrina Ask, Marie Rhodin, Maheen Rashid-Engström, Elin Hernlund, Pia Haubro Andersen

**Affiliations:** 1 Department of Anatomy, Physiology and Biochemistry, Swedish University of Agricultural Sciences, Uppsala, Sweden; 2 Univrses AB, Stockholm, Sweden

**Keywords:** Animal behaviour, Biomechanics

## Abstract

A number of facial expressions are associated with pain in horses, however, the entire display of facial activities during orthopedic pain have yet to be described. The aim of the present study was to exhaustively map changes in facial activities in eight resting horses during a progression from sound to mild and moderate degree of orthopedic pain, induced by lipopolysaccharides (LPS) administered in the tarsocrural joint. Lameness progression and regression was measured by objective gait analysis during movement, and facial activities were described by EquiFACS in video sequences (n = 348, total length 892.5 min) of the horses obtained when resting in their box stalls. Predictive modeling identified 16 action units and action descriptors, related to ears, eyes, and lower face. Lower lip depressor (AU16), lips part (AU25), half blink (AU47), single ear forward (SEAD101) and single ear rotator (SEAD104) were selected as co-occurring significantly more in horses with pain than in horses without pain. The major change in co-occurring facial activities occurred in the transition from no pain to mild pain. In conclusion, resting horses with induced orthopedic pain showed a dynamic upper and lower facial repertoire and the relationship between level of pain intensity and facial activity appears complex.

## Introduction

The horse (*Equus caballus*) is a popular companion and sports animal, but lately, concerns have been raised about equine welfare^1,2^. An important part of animal welfare assessment is deciding whether the animal is experiencing pain or not, and it is considered beneficial to include validated pain assessment tools in welfare assessment protocols^3^. Assessment of pain in horses has received considerable research attention in the past decade. Tools developed to support pain assessment include composite measure pain scales where pre-selected facial expressions, body behaviors, physical and/or physiological parameters are assessed. Lately, grimace scales solely assessing facial expressions have been developed for several animal species, including horses (Horse Grimace Scale, HGS)^4^. They contain detailed descriptions of potential pain-related facial activities, and similar facial activities are included in other equine pain scales, such as Equine Utrecht University Scale for Facial Assessment of Pain (EQUUS-FAP)^5,6^ and Equine Pain Scale (EPS)^7^. The total pain score and grimace intensity is believed to correlate with the level of pain intensity experienced by the horse, as also found in mice, where pain scores have been correlated to stimulus intensity^8^, and in humans, where level of self-reported pain is correlated with intensity of facial expressions^9^. Assessment of facial expressions using HGS and EQUUS-FAP has been found promising for moderate to severe pain in newly castrated horses^4,10^, in horses with laminitis^11^, dental problems^12^ and colic^5,6^, and in horses after head-related^13^ and orthopedic surgery^14^. For mild pain, the results vary more. The ‘equine pain face’ in EPS has been defined for mild to moderate acute short-term experimental pain induced by application of capsaicin to the skin and with a tourniquet on the antebrachium^15^. However, in horses with mild induced pain from subcutaneously polylactide-based polymer implantation, facial expressions could not detect pain^16^. In addition, observers assessing human patients with mild pain struggle to identify changes in facial expressions^17^. Thus, mild pain is generally considered difficult to identify and pre-selected grimaces may not be shown sufficiently clearly or frequently during pain assessment.

Mild orthopedic pain in horses is commonly assessed during motion through subjectively grading movement asymmetry/lameness or objectively measuring movement asymmetry. However, among e.g., riding horses and Standardbred trotters perceived as sound by their owners, up to 73% and 93%, respectively, can present with different degrees of movement asymmetry^18,19^. Equine pain scales have been used successfully for assessing moderate to severe clinical orthopedic pain in resting horses^11,14,20^, but we have previously showed that they seem to be suboptimal for assessing mild to moderate orthopedic pain at rest^21^. Identification of combinations of facial activities/grimaces not included in pain scales could therefore improve the accuracy of assessment of mild orthopedic pain. So may the understanding of how facial activities co-occur during different pain intensities. Hence, exhaustively describing, in a standardized way, all facial activities exhibited is lacking and could contribute to a more reliable assessment tool for early orthopedic pain identification.

The Facial Action Coding System (FACS)^22^ has been widely used on humans to describe facial action units present during pain and other emotions^23–25^. FACS relies on visible contractions of anatomically defined muscles that result in movement of facial structures. Facial action units are annotated by trained and certified raters, and during the annotation process presence of action units is not interpreted or related to a certain affective state. Any inference about the meaning of the facial movements is made after annotating the video. A modified FACS (EquiFACS) has been developed for horses^26^. In a previous study on capsaicin- and pressure-induced pain in a small number of horses, it was found that certain facial action units are more prevalent during pain^27^. These single action units are partly involved in the grimaces described earlier. However, the exhaustive facial repertoire displayed by horses during the progression from sound to mild and moderate degree of orthopedic pain has not been described previously.

The aim of this study was to explore changes in the facial repertoire in resting horses with different intensities of induced acute orthopedic pain. Movement asymmetry was used as a proxy for pain and to define the level of intensity. We tested the predictive performance of facial action units (AUs) and action descriptors (ADs) to identify those important for pain detection, and performed data-driven selection of co-occurring AUs and ADs under different pain intensities. The hypothesis tested that previously described AUs/ADs (half blink, ear rotator, nostril dilator, chin raiser, chewing) can predict pain and co-occur together more frequently in horses with orthopedic pain than without. We also hypothesized that AUs/ADs co-occur less frequently during mild pain than during moderate pain.

## Results

Orthopedic pain was successfully induced with intra-articular administration of lipopolysaccharides (LPS) into the hock in all eight horses. The resulting hindlimb lameness and increase in movement asymmetry were confirmed with objective gait analysis during trot. Details on changes in subjectively assessed lameness grades and in objectively measured movement asymmetry have been published elsewhere^28^. The maximum increase in movement asymmetry and the level of pain intensity varied among horses. In the data included in the present study, three horses (horse 1, 6 and 8) experienced mild pain (PD_min_ < 40 mm) based on the maximum increase in movement asymmetry. Four horses (horse 2, 4, 5 and 7) experienced both mild and moderate pain, and horse 4 experienced only moderate pain based on the maximum increase in movement asymmetry (PD_min_ > 40 mm). Rescue protocol was initiated in two horses, providing adequate pain relief after evacuation of synovia. Moreover, pain was assessed with pain scales during rest, and total pain scores for each pain assessment have been published elsewhere^21^. A total of 348 video recordings during the pain assessments were annotated with EquiFACS. The total length of annotated video sequences was 892.5 min and the dataset contained 37,072 annotations, with 20,208 annotations in ‘no pain’ sequences and 16,864 annotations in ‘pain’ sequences. Every annotation represents the start and stop time for one AU or AD, meaning that the dataset in total contained 37,072 facial activities. Descriptive statistics are presented in Table 1 and the frequency distribution of facial activities per horse and face region can be found in Supplementary Material (S1 Figure).

**Table 1:** Descriptive statistics on the annotation data set. Each annotation represents one specific facial activity. Median, and 1st and 3rd interquartile for the number of annotations per video sequence, the number of annotations per horse and the duration of each annotation in seconds, in video sequences labeled as ‘no pain’ and ‘pain’.

Descriptive statistics	Median (1st and 3rd interquartile)	
	No pain	Pain
Number of annotations per video	102.5 (65.8, 151.3)	84.0 (61.0–129.0)
Number of annotations per horse	2314.0 (1822.0, 3267.25)	1997.5 (1259.0, 2308.0)
Duration of annotations (s)	0.466 (0.340, 0.700)	0.491 (0.350, 0.750)

### Identification of AUs and ADs associated with orthopedic pain

**Figure 1:**
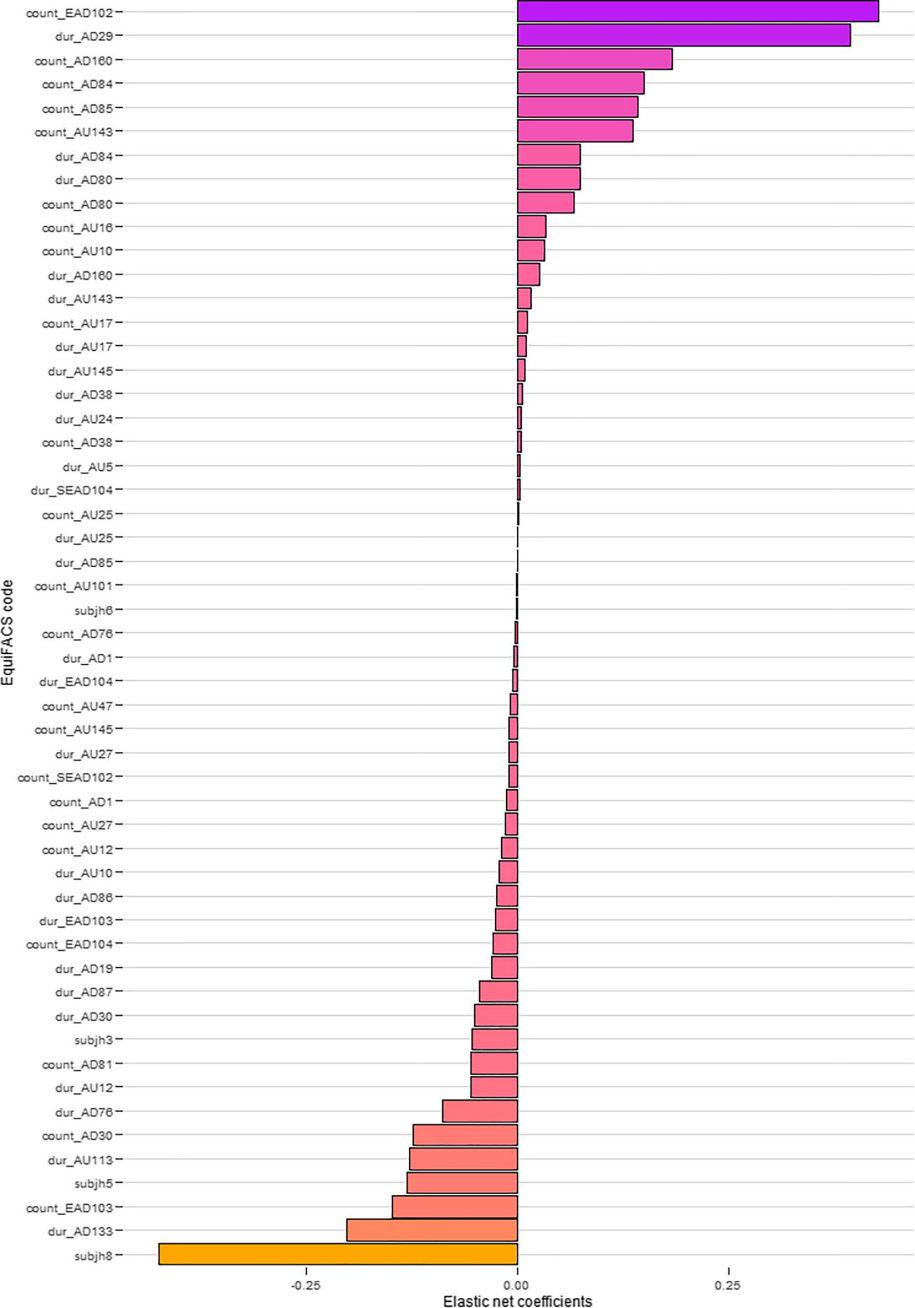
Regressions coefficients in elastic net regression testing the predictive performance of action units (AUs) and action descriptors (ADs). The frequency (‘count’) and duration (‘dur’) of each EquiFACS code are shown. Positive coefficient values indicate an association with presence of pain.

Whether the EquiFACS codes could predict the presence of ‘pain’ or the presence of ‘no pain’ was tested with elastic net regression. Model coefficients are presented in S1 File and plotted in Fig. 1. Those with a coefficient of 0 were considered unable to predict pain. The best predictor for pain chosen by the model was frequent adduction of both ears (EAD102), and duration of one ear rotating (SEAD104) was a weak predictor of pain. Most other ear-related codes were not able to predict pain. Frequent and a long duration of both ears flattening (EAD103) and rotating (EAD104), and frequent adduction of one ear (SEAD102) were associated with no pain. Out of the codes belonging to the upper face, frequency and duration of *eye closure* (AU143), and duration of *blink* (AU145) and *upper lid raiser* (AU5) were classified as predictors of pain. Twelve out of 23 predictors associated with pain were AUs/ADs belonging to the lower face: duration of *lower jaw thrust* (AD29), frequency and duration of *lower lip relax* (AD160), frequency of *lower lip depressor* (AU16), frequency of *upper lip raiser* (AU10), frequency and duration of *chin raiser* (AU17), duration of *lip presser* (AU24), frequency and duration of *nostril dilator* (AD38), and frequency and duration of *lips part* (AU25). In addition, frequent and long duration of *head shake side to side* (AD84), frequent *head nod up and down* (AD85), and frequency and duration of *swallowing* (AD80) were relatively strong predictors of pain. The AUs/ADs associated with no pain were mainly action descriptors: duration of *blow* (AD133), *ear shake* (AD87), *tongue show* (AD19), *grooming* (AD86), *head nod up and down* (AD85), and *chewing* (AD81), and frequency and duration of *jaw sideways* (AD30), *yawning* (AD76), and *eye white increase* (AD1). Duration of *sharp lip puller* (AU113), *lip corner puller* (AU12) and *upper lip raiser* (AU10), frequency and duration of *mouth stretch* (AU27), and frequent *inner brow raiser* (AU101), *half blink* (AU47) and *blink* (AU145) were also associated with no pain. Furthermore, horse 3, 5, 6 and 8 were identified as negative predictors of pain in the model.

### Co-occurrence of AUs and ADs during different levels of pain intensity

**Figure 2:**
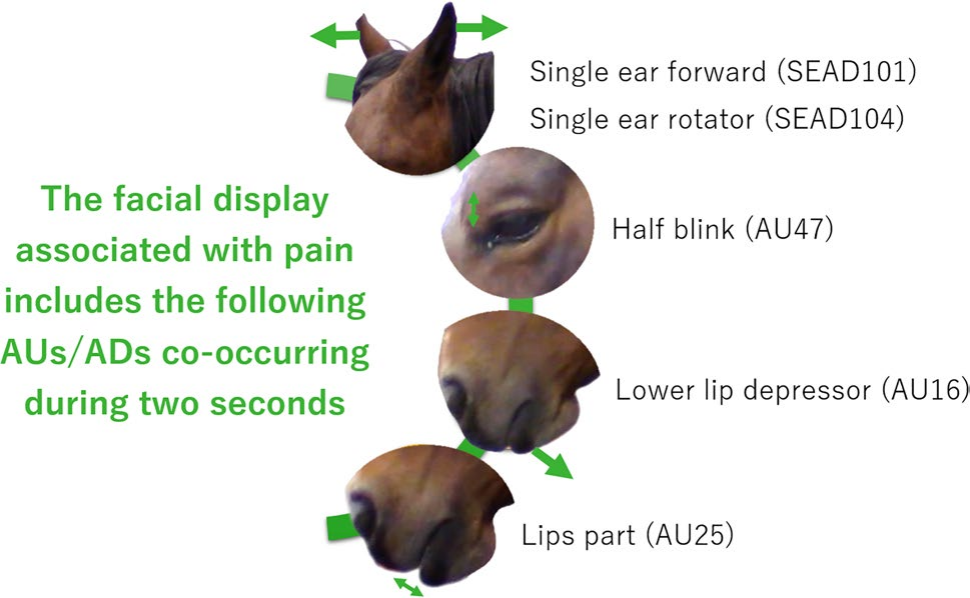
EquiFACS codes selected by the co-occurrence method and co-occurring significantly more in ‘pain’ than in ‘no pain’ video sequences (p < 0.05). Observation window size was 2 s.

On comparing ‘no pain’ video sequences with ‘pain’ sequences, the co-occurrence method identified the following AUs and ADs as conjoined: *lower lip depressor* (AU16), *lips part* (AU25), *half blink* (AU47), *single ear forward* (SEAD101), and *single ear rotator* (EAD104) (Fig. 2). AUs and ADs selected by the co-occurrence method for the different levels of pain intensity (defined by movement asymmetry) are presented in Table 2.

**Table 2:** Action units (AUs) and ear action descriptors (EADs) selected by the co-occurrence method. Pain intensity levels were defined based on movement asymmetry. Not all horses experienced both levels of pain intensity, why the number of horses (n) experiencing each intensity level is stated in brackets. The frequency of co-occurring AUs and ADs in ‘no pain’ video sequences was compared to the frequency in ‘mild pain intensity’ sequences (0–1) and ‘moderate pain intensity’ sequences (0–2). The frequency of co-occurring AUs and ADs in ‘mild pain intensity’ sequences was also compared to the frequency in ‘moderate pain intensity’ sequences (1–2). The observation window size (OWS) was set to 2 s. p-values are shown in brackets (paired t-test) and differences were considered significant at p < 0.05.

EquiFACS code	Level of pain intensity		
	0–1 (n = 7)	0–2 (n = 5)	1–2 (n = 4)
Ears			
*Single ear forward* (SEAD101)	✓ (p < 0.001)		
*Single ear rotator* (SEAD104)	✓ (p < 0.001)	✓ (p < 0.001)	
Upper face			
*Blink* (AU145)	✓ (p < 0.001)		
*Half blink* (AU47)	✓ (p < 0.001)	✓ (p < 0.001)	✓ (p < 0.001)
Lower face			
*Upper lip raiser* (AU10)	✓ (p = 0.210)		
*Lower lip depressor* (AU16)	✓ (p = 0.241)		
*Lips part* (AU25)	✓ (p = 0.753)		

## Discussion

Resting horses experiencing orthopedic pain show a dynamic facial repertoire consisting of asymmetrical ears, and changes in facial activity in the eye area and lower face. To our knowledge, this is the first study to use EquiFACS and movement asymmetry as outcome measures for studying pain in horses. Both these methods are considered highly objective methods, compared to the visual assessment of lameness and pain. Using these objective methods, it was possible to corroborate some of the earlier findings on facial expressions during pain in horses, such as the presence of asymmetrical ears during pain and the importance of half blink in pain detection. We also identified in more detail other features such as the dynamic lower facial activities during pain, compared to previous studies showing a more stoic lower face. The present study demonstrated that predictive modeling can identify AUs and ADs associated with orthopedic pain, where several AUs and ADs related to the ears, lower face, and eyes, were identified at the peak of pain intensity. From a statistical point of view, when these AUs/ADs occur frequently and for a long duration compared with during baseline, they can indicate pain. However, when assessing pain under clinical settings, the veterinary surgeon rarely knows the baseline state of the equine patient, so it might be difficult to extrapolate these predictions into clinical pain assessments. Therefore, we added the co-occurrence method^27^ to our predictive modeling. With this method, we were able to obtain more information on AUs and ADs that occur together during different pain intensities, which may be more clinically relevant.

To increase the probability of horses showing a facial repertoire related to pain, we included video sequences from time points when each horse was assessed to be at its highest level of pain intensity. Pain assessment was performed with CPS, the pain scale found to perform best in assessing orthopedic pain in resting horses^21^. Importantly, it does not include assessment of facial expressions, so biased selection of video sequences containing pre-selected grimaces was avoided. Still, it is unlikely that pain-related facial expressions are shown constantly. In videos of horses experiencing pain, typically only 6.1% of the frames contain three or more pain-specific AUs^27^, and thus using longer duration of video sequences for annotations and pain assessments is important^29^. Therefore, annotated video sequences in the present study ranged from two to three minutes in length, resulting in an extensive total length of annotated video material of almost 900 min. This is longer than in other previous equine studies using EquiFACS^27,30^ and in the majority of studies on human pain^31^. The reason for not including even longer video sequences was the very time-consuming process of annotating, although more annotated video material would be beneficial for understanding how the facial repertoire varies during pain.

Orthopedic pain was best predicted with the help of *ear adductor* (EAD102) (Fig. 2), where “the ear(s) are pulled towards the midline” and “the distance between the ears decreases”^26^. This have not been described before and contradicts the increased distance between the bases of ears defined as “lowered ears” in the equine pain face^15^. Lowered ears are difficult to describe with EquiFACS since it probably occurs due to relaxation of muscles overlaying the parietal bone with concomitant outward rotation, but the most correct coding would be the action descriptor *ear rotator* (EAD104) and maybe even *ear flattener* (EAD103) describing ear abduction in combination with pulling the ears closer to the neck^26^. We found that high frequencies and long duration of both ears flattening and rotating (EAD103 and EAD104) instead were associated with no pain, which is in agreement with previous findings that lowered ears can be seen during sleep^15^. This further hampers the interpretation of the presence of lowered ears, unless they co-occur with other AUs. Our multiple camera set-up that recorded horses moving freely in their box stalls, might have yielded greater variation in ear movements than in horses standing still and allowed us to record the horses from the front, where *ear adductor* (EAD102) is much easier to annotate. In fact, if horses frequently switched between lowered ears and other positions, *ear adduction* (EAD102) might be coded as the ears are brought closer to each other when muscles are contracting to move the ears. We also demonstrated that long duration of one ear rotating (SEAD104) is associated with pain, and that *single ear forward* (SEAD101) and *single ear rotator* (SEAD104) co-occurred significantly more during pain. This in combination with frequently adducted ears may constitute the “asymmetrical ears” described in the equine pain face^15^. The ear position is commonly assessed in pain scales and described as ears being “stiffly backwards” in HGS^4^, or ears having a “delayed/reduced response to sound” and “position: backwards/no response to sounds” in EQUUS-FAP^5,6^. However, the present study show that ears are dynamic during pain in freely moving horses, where asymmetrical ears may contribute more to the pain detection than both ears backwards. Importantly, ear movements co-occurred together with other AUs/ADs indicating that the ear position should not be assessed alone, especially since flattening and rotating both ears (EAD103 and EAD104) were associated with a no pain state in this study. Additionally, since these changes in ear position can be rapid, small and difficult to observe in real-time, interpretation of ear movements from still images seems impossible. One should also be careful about relying only on ear movements visible to the eye when assessing pain in horses.

Several eye-related AUs/ADs were able to predict pain in the present study, confirming previous findings on eye-related facial expressions during pain. Closing the eye frequently and for a long time (AU143), making long blinks (AU145), and raising the upper eyelid (AU5) frequently and for a long time were associated with pain. In addition, *blink* (AU145) significantly co-occurred with other AUs/ADs during mild pain, and have been associated with pain previously—as have *upper lid raiser* (AU5)^27^. *Eye closure* (AU143) has not, but may be represented by “orbital tightening” in HGS^4^, supporting its importance in pain recognition. *Upper lid raiser* (AU5) may result in exposing the sclera, so *eye white increase* (AD1) was an anticipated predictor of pain. *Eye white increase* (AD1) and *inner brow raiser* (AU101) are empirically associated with pain and included in the facial configurations in existing pain scales^4–6,15^, but in the present study they were unable to predict pain, which corroborates previous inconsistencies in results^27^. *Half blink* (AU47) did not successfully predict pain in the present study, in fact, frequent *half blink* (AU47) predicted a ‘no pain’ state. However, it co-occurred significantly with other AUs/ADs during all levels of pain intensity, which is in agreement with Rashid et al.^27^. Hence, when *half blink* (AU47) co-occurs with asymmetrical ears and lower face activity, the horse is most probable in pain. As with ear movements, eyelid movements (blinking, half blinks, and raising the upper eyelid) are difficult to observe in real time, despite their association to pain. In the present study, *eye closure* (AU143) is a strong predictor of pain and can be easier to observe in real-time, why it may be the most clinically relevant predictor of pain among the eye-related AUs/ADs. In the present study, frequency and duration of *eye white increase* (AD1), and frequent *inner brow raiser* (AU101) and *blink* (AU145) were associated with no pain. These codes have previously been identified as co-occurring during stress^30^ and may indicate that the horses in the present study experienced stress during the ‘no pain’ state, despite thorough acclimatization prior to the experiment. Moreover, experiencing pain is an internal stressor that cannot be avoided^32^, why the horses probably experienced stress during the ‘pain’ state as well. Hence, the study of pain in animals is often challenged by the potential co-presence of stress, why a combination of stress-related and pain-related facial activities may be shown by the horse during an assessment and confuse the pain assessment and the results. It can therefore be theorized that pain assessment in horses would benefit from considering stress expressions, since stress-related facial activities might conceal pain-related facial activities. For this to be possible, it is critical to identify how stress and pain interact and how stress influences the expression of pain.

The majority of codes with positive prediction coefficients belonged to the lower face. Duration of *jaw thrust* (AD29), described as when “the lower jaw is pushed forward” or “the lower teeth extend in front of the upper teeth”^26^ had the biggest coefficient out of codes belonging to the lower face. On inspecting the dataset, however, we found that it had only been annotated three times among the 37,072 annotations, and that it had a longer duration in ‘pain’ sequences than in ‘no pain’ sequences. This may explain its predictive value identified in the elastic net regression model (Fig. 2). However, *jaw thrust* (AD29) has previously been selected by the co-occurrence method in horses with clinical pain^27^. Thus, the importance of *jaw thrust* (AD29) in pain recognition should be further explored. Frequency and duration of *lower lip relax* (AD160) were also rather strong predictors of pain, which seems odd from a clinical perspective as a horse experiencing pain should not have a relaxed lower lip according to previous studies on pain-related facial expressions^4,15^. Inspection of the dataset revealed that *lower lip relax* (AD160) had been annotated 11 times, in both ‘no pain’ and ‘pain’ sequences, although in one ‘pain’ sequence it was annotated for 41 s. This most probably explains the association to pain in the model. Hence, *lower lip relax* (AD160) should not be used as a predictor for pain. Frequency of *upper lip raiser* (AU10) and *lower lip depressor* (AU16), frequency and duration of *chin raiser* (AU17), *nostril dilator* (AD38) and *lips part* (AU25), and duration of *lip pucker* (AU18) and *lip presser* (AU24), were all predictors of pain. *Chin raiser* (AU17) and *nostril dilator* (AD38) are lower facial configurations described in HGS^4^, EQUUS-FAP^5,6^, and the equine pain face in EPS^15^, and have previously been identified as strong positive predictors for pain when seen together within 10–15 s^27^. Our results confirm this, where occurring frequently and during a long time seem to be associated with pain. However, they did not co-occur more in pain sequences in the present study, why more research is needed on how *nostril dilator* (AD38) and *chin raiser* (AU17) are co-occurring during different types of pain and intensities. According to EquiFACS, frequently raising the upper lip (AU10) and pulling the lower lip down ventrally or laterally (AU16) are very often coded together with the lips being part (AU25)^26^. This was reflected in the predictive model assigning high predictive values to these AUs, and in the co-occurrence method where *lower lip depressor* (AU16) and *lips part* (AU25) co-occurred during pain. Additionally, *upper lip raiser* (AU10) co-occurred with the two others during mild pain. *Upper lip raiser* (AU10) has previously been identified as a conjoined AU, leading to a suggestion that lower facial activity should be considered an indicator of pain^27^. The opposite to mouth opening, i.e., pressing the lips together for a long time (AU24), also seemed to be associated with pain, and is in fact included in the equine pain face in EPS^7,15^. Thus, frequently separating the lips and moving the upper and lower lips may be indicative of pain, as may pressing the lips together for a long time. This ‘mouth-playing’, i.e., a combination of many small movements involving the mouth, has been described as a gross pain behavior in EPS^7^, but has not been considered to be related to mild and moderate pain. Other mouth-related behaviors are *flehmen* and *yawning*, described in both EPS^7^ and EQUUS-FAP^5^ and *chewing* (AD81) identified as conjoined previously^27^. Changes in lower facial expressions have been demonstrated in horses during pain, but more stoic and constant looks are described (‘mouth strained and pronounced chin’^4^, ‘edged shape of the muzzle with lips pressed together’^15^ and ‘slightly/obviously lifted corners mouth/lips’^5^). The present study is the first to show the dynamics of the facial repertoire, since exhaustively coding lower facial activity with EquiFACS in longer video sequences (> 2 min) allowed more dynamic mouth behavior to emerge.

Furthermore, we identified variations in AUs/ADs in relation to the level of pain intensity in horses (Table 2). Using movement asymmetry to define the pain intensity level, it seems like horses experiencing mild pain had more codes co-occurring than horses experiencing moderate pain. This is an interesting finding, questioning previous theories on synchronized increase assumptions on positive and linear correlation between numbers of facial expression and pain intensity. If the major change in facial activity actually take place when the horse goes from experiencing no pain to experiencing mild pain, it might be misleading to approximate the level of pain intensity from the presence of facial expressions. According to the present results, only *half blink* (AU47) in combination with *single ear rotator* (SEAD104) may indicate that the horse experiences moderate pain. One explanation might be that horses perhaps perform more pain-related body behavior during moderate pain, such as postural changes, and that they result in pain relief. This was discussed by Ask et al.^28^ on finding that facial expressions was seen infrequently together with postural changes in horses with orthopedic pain. In addition, the low sample size in the present study may affect the results, since pain is a highly individual experience that might vary in how it is expressed among individuals. Possibly, there is a greater variation in how facial activities co-occur during moderate pain, resulting in a need for a bigger sample representing more intensity levels to identify differences in their co-occurrence. The definition of mild and moderate pain intensity was based on the objectively measured increase in movement asymmetry rather than subjective lameness grades. However, the relation between increasing movement asymmetry during pain (thereby also increasing lameness grade), and the pain intensity experienced by the horse may not be linearly correlated. Thus, for two horses with an increase in movement asymmetry of 40, one may experience moderate pain and the other one mild pain. For future studies, further objective measures that can act as proxies for pain should therefore be included. There might be an interesting time aspect as well. Some horses may choose to seek more contact with the observer when they experience that pain is increasing more and more, thus, affecting the co-occurrence of facial activities. Gleerup et al.^15^ noticed that pain-related facial expressions were less pronounced when horses interacted with the observer, and that contact seeking may be an early sign of pain. The presence of different facial repertoires during different levels of pain intensity illustrates the intricacy of pain intensity, and implies that there may not be a linear correlation between a grimace being present and the intensity. Hence, the concept of one prototypical pain face may be a simplification and further studies are needed on the relationship between pain intensity and facial repertoire.

To study the relationship between intensity and facial activity, intensity scores could be added to individual EquiFACS codes, as it is done for FACS in humans^22^. The definition of the level of pain intensity in FACS is done by adding subjective intensity scores of 1–5 when coding *brow-lowering* (AU4), *cheek-raising* (AU6), *eyelid tightening* (AU7), *nose wrinkling* (AU9), *upper-lip raising* (AU10), *oblique lip raising* (AU12), and *horizontal lip stretch* (AU20)^9^. Intensity score 1 indicates that only traces of the AU are present and intensity score 5 that the AU is present to the maximum. The scores of all AUs are then combined to estimate the level of pain. Since our results indicated that only a few facial AUs are present during mild pain, it would be interesting to explore this further. In EquiFACS, minimum criteria exist for coding one AU, but not intensity scores^26^. Hence, there might be a risk that certain AUs performed with very low intensity will not be annotated with EquiFACS if the minimum criterion is too high.

A limitation of the present study is that annotated video sequences were recorded in the presence of observers, because it is known that observer presence may cause horses to disrupt discomfort behaviors^33^. It is believed that pain-related facial expressions are more difficult to “hide” than body behaviors, but it is not known whether observer presence might result in the horse trying to conceal facial expressions or not. Including video sequences without the presence of observers could perhaps have resulted in annotations that were more representative of the level of pain intensity, but in our opinion annotating sequences in the presence of observers is more clinically relevant. Hence, clinicians performing live pain assessments of horses in pain can expect AUs/ADs identified in this study to be present when horses are assessed at the clinic. Due to ethical concerns about inducing pain, only eight horses were included in the present study. This is in concordance with the sample size of previous studies^34–45^, where one type of pain was induced by an inflammatory insult, LPS in six to eight horses. On the other hand, a low sample size and/or low study power may affect the outcomes of the study with results difficult to interpret. Therefore, well aware of the risk of highly individual variations in facial activity, we included ‘horse’ (each individual) as a fixed effect in the predictive modeling statistics to identify activities occurring due to the individual variation rather than the presence of pain. As a result, four of the horses turned out to be associated with a no pain state, i.e. confirming that there are some individual variation in facial activities during the no pain states. We followed the full course of pain from induction, increase, peak, resolution, resulting in increased movement asymmetry and pain behavior in all horses, which is concordant with findings in previous studies^34,35,38^. If spontaneously lame horses are used, difficulties arise in deciding when pain is present or not and in standardizing the level of pain intensity. For instance, horses with clinical lameness may be more prone to experience chronic pain with gradually evolving lameness or lameness that is difficult to treat. In addition, pain-related facial expressions may be less prominent or different in horses with chronic pain^46^, but this needs further investigation.

In conclusion, the present study identified sixteen AUs and ADs important for pain recognition using predictive modeling and the co-occurrence method based on annotations of > 2 min video sequences of horses with induced orthopedic pain. These were: *ear adductor* (EAD102), *jaw thrust* (AD29), *head shake side to side* (AD84), *head nod up and down* (AD85), *eye closure* (AU143), *swallowing* (AD80), *lower lip depressor* (AU16), *upper lip raiser* (AU10), *chin raiser* (AU17), *blink* (AU145), *upper lid raiser* (AU5), *lip presser* (AU24), *nostril dilator* (AD38), *single ear rotator* (SEAD104), *lip pucker* (AU18), and *lips part* (AU25). The co-occurrence method selected *lower lip depressor* (AU16), *lips part* (AU25), *half blink* (AU47), *single ear forward* (SEAD101) and *single ear rotator* (SEAD104) as co-occurring significantly more in horses experiencing pain. Hence, resting horses with induced orthopedic pain show a dynamic upper and lower facial repertoire, characterized by asymmetrical ears, facial activity in the eye area, and ‘mouth-playing’. The present study is the first to show changes in facial activity in relation to level of pain intensity. The majority of changes in facial activity occurred in the transition from no pain to mild pain. This is not in accordance with the hypothesis, and requires further research to clarify the relationship between intensity and facial activity. However, the present results highlight that the concept of one prototypical pain face may be a simplification of the full pain-related facial repertoire.

## Methods

The study protocol was approved by the Swedish Ethics Committee (diary number 5.8.18-09822/2018) in accordance with Swedish legislation on animal experiments. Replacement, reduction, and refinement were considered in depth and the ARRIVE guidelines were followed^47^. The study was designed to generate data for several purposes.

### Animals and study design

Detailed information about the study design has been published elsewhere^28^. In summary, seven Standardbred trotters and one Warmblood horse (six mares and two geldings, mean (standard deviation, SD) age 14.5 (3.7) years, body mass 552 (39) kg, and height at withers 160 (2.8) cm) were recruited for the study. The horses were clinically healthy and ≤ 1 grade lame on a 0–5 ordinal lameness scale ranging from 0 = sound, to 5 = non-weight bearing^48^. Grade 3 represents a moderate lameness visible during straight line trot. After 10–12 days of acclimatization, objective gait analyses and pain assessments were performed to measure baseline movement asymmetry and pain scores. Transient aseptic inflammatory arthritis was then induced in accordance with previous substantial research on this method^38^ in the hindlimb with the highest baseline movement asymmetry, resulting in fully reversible orthopedic pain of mild to moderate intensity over 0–52 h. To induce arthritis, 3 mL of a diluted ready-made solution (*L5418 Sigma,* 1.167 ng/mL) of lipopolysaccharides (LPS) from *E. coli* O55:B55 were injected into the tarsocrural joint using routine clinical and aseptic techniques. After 1.5 h of resting in the box stall, a minimum of four objective gait analyses and pain assessments were performed until each horse returned to a movement asymmetry comparable to that at baseline (before lameness induction). Rescue analgesia by evacuation of synovia according to clinical standard procedures was performed if the lameness exceeded 3/5 grades.

### Objective gait analysis and assessment of pain

For each objective gait analysis, each horse was equipped with seven skin-mounted spherical markers (38 mm diameter, Qualisys AB, Gothenburg, Sweden), and walked and trotted in a straight line on hard and soft surfaces. Lunging was performed on a soft surface. Marker positions were recorded by 13 infrared optical motion capture cameras (Qualisys AB, Gothenburg, Sweden) operating at 200 Hz, tracked by QTM software (version 2.11-2019.3, Qualisys AB, Gothenburg, Sweden), and images were visually inspected before exporting and analyzing the data in MatLab^49^. Details of the filtering process and stride segmentation can be found in our previous publications^50,51^. For the purposes of this study, vertical displacement data during trot in straight line on a hard surface were collected from markers placed on the poll of the head and tubera sacrale of the pelvis. From the vertical displacement signal two movement asymmetry metrics were calculated which quantify the difference in minimum height of head (HD_min_) and pelvis (PD_min_) during the left and right stance phase of each stride. These metrics are commonly used to describe weight-bearing hindlimb lameness (PD_min_) and related compensatory patterns (HD_min_)^48,52^.

The days prior to induction, baseline objective gait analysis and pain assessments were performed. Another pain assessment was performed on the morning of the induction, resulting in three occasions representing a no pain state. After induction, pain was assessed when the horse was resting in its box stall before and after objective gait analyses, and sometimes also between objective gait analyses. Every assessment included 2-min pain scorings using the pain scales HGS, EQUUS-FAP, and EPS and a 5-min pain scoring with the Composite Orthopedic Pain Scale (CPS)^20^, in that order. Three observers performed the assessments independently and simultaneously from outside the box stall.

### Video recording

Prior to arrival of the horses, four infrared network surveillance cameras (WDR ExIR Turret Network Camera, Hikvision Digital Technology Co., Hangzhou, China) were installed on the wall or the bars in each box stall corner, at a height of approximately 180 cm. Nine lamps were attached to the ceiling in each box stall, at a spacing of 75 cm, to avoid shading of the face during video recording. Strip lighting (cold white, 18W, 4000 Kelvin) was used, and was automatically turned on and off every morning and night with a plug-in timer. Continuous recording commenced 12 h before baseline measurements and ended after the final measurement. Recordings from each of the four surveillance cameras were exported as large .mp4-files that had to be edited into shorter video sequences.

### Selection of video sequences for annotation

**Figure 3:**
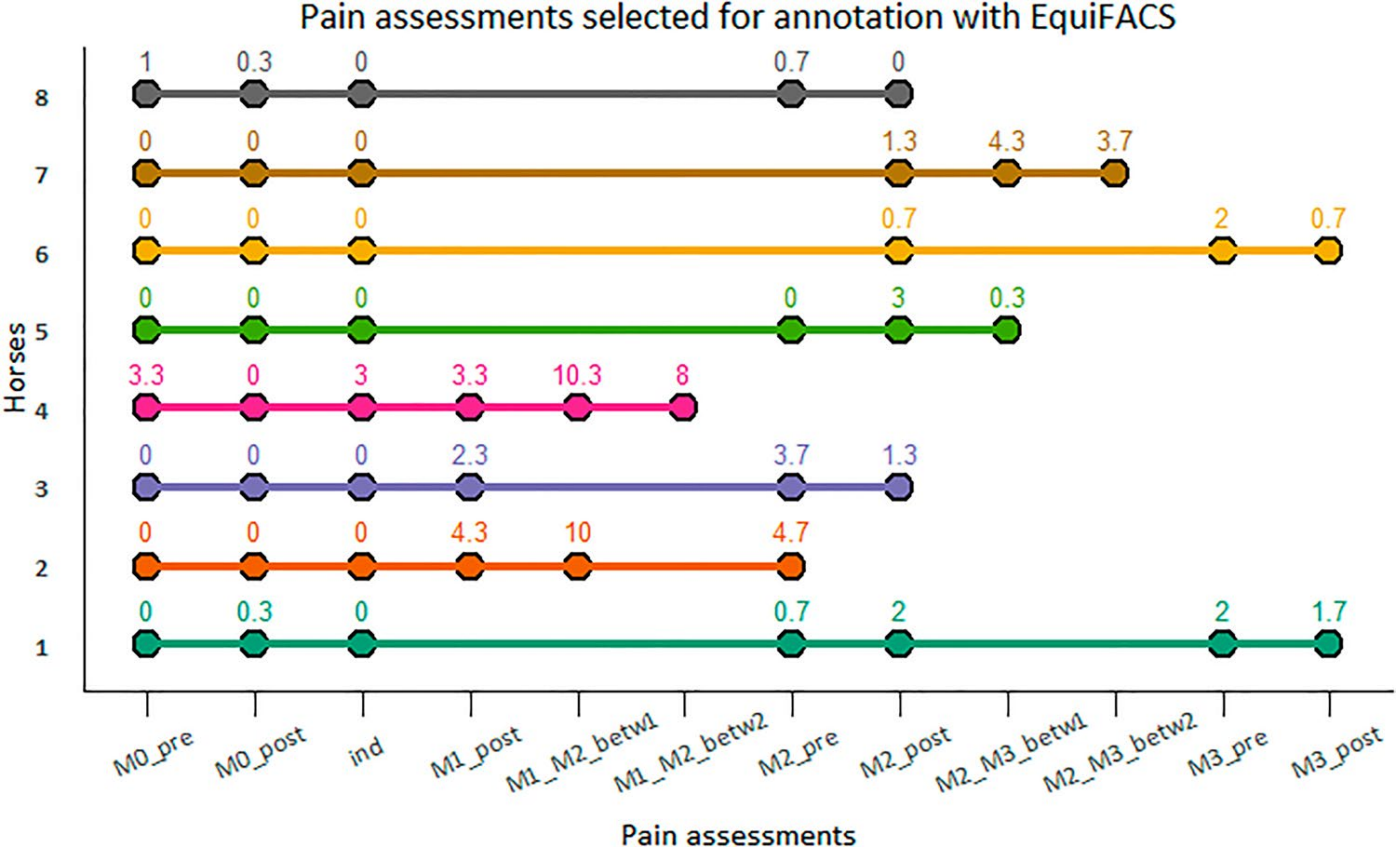
Pain assessments selected for annotation for each horse. Horses 1–8 are listed on the y-axis and pain assessments on the x-axis. ‘M0_pre’ (pain assessment before the baseline objective gait analysis), ‘M0_post’ (pain assessment after the baseline objective gait analysis), and ‘ind’ (pain assessment on the morning of the induction) represent ‘no pain’, while remaining assessments represent ‘pain’. The objective gait analyses are designated M0-M3, ‘pre’ or ‘post’ indicates whether the assessment was performed directly before or after objective gait analysis, and ‘betw1’ and ‘betw2’ refer to assessments performed between objective gait analyses. Mean Composite Orthopedic Pain Scale (CPS) total pain scores are stated for each point in the graph.

Three pain assessments performed prior to induction were selected to represent a ‘no pain’ state. Pain assessments representing a ‘pain’ state were selected based on pain scores from CPS, a pain scale with known performance parameters^21^. The assessment with the highest mean CPS total pain score and the assessments immediately before and after were included, resulting in 48 pain assessments (Fig. 3), where each pain assessment contained four pain scorings (one for every pain scale). Video recordings from these were cut out manually from the large .mp4-files, resulting in 192 sequences for each camera angle and 768 sequences in total. Video sequences containing pain scorings with HGS, EQUUS-FAP, and EPS were each about two minutes long. Those of CPS were about three minutes long since the last two minutes of the five-minute scoring time contained human interaction with the horse to measure physiological parameters, and were excluded. The 768 video sequences were then run through automated horse face detection software^53^ to select the videos from the two best camera angles for each pain scoring. This resulted in 384 sequences ready for annotation.

### Annotations with EquiFACS

All video sequences were blinded, renamed, and randomly distributed among nine EquiFACS-certified^26^ annotators (four equine veterinarians, one agronomist, one agronomy student, two veterinary students, and one veterinary nursing student). Annotations were carried out in ELAN Linguistic Annotator^54^ and video sequences were viewed in normal speed and frame-by-frame during the annotation process. Onset and offset of movement of facial structures were annotated, and AUs or ADs were assigned to the facial movements identified. A modified annotation template, where head movement and visibility codes were excluded, was used (S1 Table). The names of all AUs and ADs are included in the text and in S1 Table. A new code called ‘unscorable face’ was used when the whole face was out of camera view, blurry, or shaded. To assess inter-observer agreement, all annotators annotated the same sequence once without knowing. This sequence depicted M2_post of horse 6 (Fig. 3). Inter-observer agreement was computed as described by Ekman et al.^22^, using the Wexler ratio. There was good inter-observer agreement between the nine observers, with mean (SD) agreement of 0.85 (0.05).

### Data processing and statistical analyses

Annotations were merged into one dataset and subjected to further cleaning. Two annotated files were removed since they were not annotated completely. If > 70% of a video sequence was annotated with ‘unscorable face’, it was excluded from the dataset. This resulted in exclusion of 34 video sequences (16 ‘no pain’, 18 ‘pain’), and the remaining 348 video sequences (176 ‘no pain’, 172 ‘pain’) were used for statistical analysis. Total duration and frequency of each annotation per video sequence were included in the dataset, resulting in 78 variables (39 variables for annotation duration and 39 for annotation frequency). In addition, pain intensity labels (no pain (0), mild pain (1), and moderate pain (2)) were added to the dataset to describe variations in the level of pain experienced by the horses. The level of pain intensity was defined based on increase in movement asymmetry, where the ‘no pain’ label (0) was automatically assigned to video sequences recorded before induction. An increase in PD_min_ < 40 mm was taken to represent mild pain (1), while an increase in PD_min_ > 40 mm was taken to represent moderate pain (2). Video-sequences of pain assessments performed between objective gait analyses (Fig. 3) represented moderate pain (2). The labeling system is presented in S1 File.

Descriptive statistics and regularized regression modeling were carried out in R^55^. Shapiro Wilks test (p < 0.05) and histograms indicated non-normality in the dataset, so median and 1st and 3rd interquartile were computed for number of annotations per video sequence, number of annotations per horse, and duration of annotations. Distributions of annotations were plotted with ‘ggplot2’^56^. Outliers were included in the dataset. To test predictive performance of the 78 variables in the dataset, where the presence of pain (0/1) was the outcome, regularized logistic regression modeling was performed instead of regression modeling, where the maximum likelihood is estimated. Regularization involves penalty terms, the hyper-parameters alpha and lambda, for including a high number of variables in the model, but reduces variation in the model and thereby improves the predictive performance. Due to highly correlated variables, an elastic net regression model was fitted with ‘caret’^57^, allowing for tuning of alpha and lambda. Tuning was done with tenfold cross-validation, resulting in alpha = 0.1 and lambda = 0 for the model with the highest predictive performance (described by area under the curve (AUC) = 0.654). Horse (subj1-8) was included as a fixed effect to account for outcomes that could be explained by the individual horse. Model coefficients were then plotted with ‘ggplot2’.

To identify AUs and ADs co-occurring together during different levels of pain intensity, the existing co-occurrence method was used^27^. This method performs data-driven selection of AUs and ADs occurring together during given pain states, and a paired t-test is used to determine if the co-occurrence is significantly different between the compared pain states (p < 0.05). ‘No pain’ video sequences were compared with ‘pain’ sequences to test which AUs/ADs that occurred together during pain. Comparisons were also made between each level of pain intensity defined by movement asymmetry. The threshold for selecting AUs/ADs (α) was set to 0.5 in the model. Observation window size (OWS) was set to 2 s and defined when AUs/ADs were considered to be co-occurring. If co-occurring in the same OWS and occurring significantly more frequently, the AUs and ADs were referred to as conjoined. Conjoined AUs and ADs for OWS = 2 were the main interest, since AUs and ADs in a short OWS seem to appear coherently to the eye, hence generating a grimace.

### Supplementary Information

Supplementary Information 1.

Supplementary Information 2.

## Data Availability

Data used for descriptive statistics and predictive modeling are provided in the supplementary materials. The raw annotation dataset is available from the corresponding author (KA) on reasonable request.
