# Supplementary material for: Changes in the equine facial repertoire during different orthopedic pain intensities
Source: Sci Rep. 2024 Jan 2;14:129. doi: 10.1038/s41598-023-50383-y (PMC10762010; doi:10.1038/s41598-023-50383-y)

Supplementary material

S1 File. Excel spreadsheet (in a separate Excel file) with an overview of the data, EquiFACS annotations, and output and coefficients from the elastic net regression modeling.

S1 Table. Modified ELAN template for annotation with EquiFACS

Tier	AU/AD	Meaning
**Ears**	EAD101 (R/L)	*Ear forward*
	EAD102 (R/L)	*Ear adductor*
	EAD103 (R/L)	*Ear flattener*
	EAD104 (R/L)	*Ear rotator*
	Unscorable (R/L)	*Ear not visible*
**Upper face**	AU101	*Inner brow raiser*
	AU143	*Eye closure*
	AU145	*Blink*
	AU47	*Half blink*
	AU5	*Upper lid raiser*
	AD1	*Eye white increase*
**Lower face**	AU10	*Upper lip raiser*
	AU12	*Lip corner puller*
	AU113	*Sharp lip puller*
	AUH13	*Nostril lift*
	AU16	*Lower lip depressor*
	AU17	*Chin raiser*
	AU18	*Lip pucker*
	AU122	*Upper lip curl*
	AU24	*Lip presser*
	AU25	*Lips part*
	AU26	*Jaw drop*
	AU 27	*Mouth stretch*
	AD38	*Nostril dilator*
	AD160	*Lower lip relax*
**Miscellaneous**	AD19	*Tongue show*
	AD29	*Jaw thrust*
	AD30	*Jaws sideways*
	AD133	*Blow*
**Gross behaviour**	AD50	*Vocalization*
	AD76	*Yawning*
	AD80	*Swallow*
	AD81	*Chewing*
	AD84	*Head shake side to side*
	AD85	*Head nod up and down*
	AD86	*Grooming*
	AD87	*Ear shake*
**Unscorable face**	Unscorable	*Face not visible (excl. the ears)*

S1 Figure. Distribution of annotation data between ‘no pain’ and ‘pain’ videos per horse.

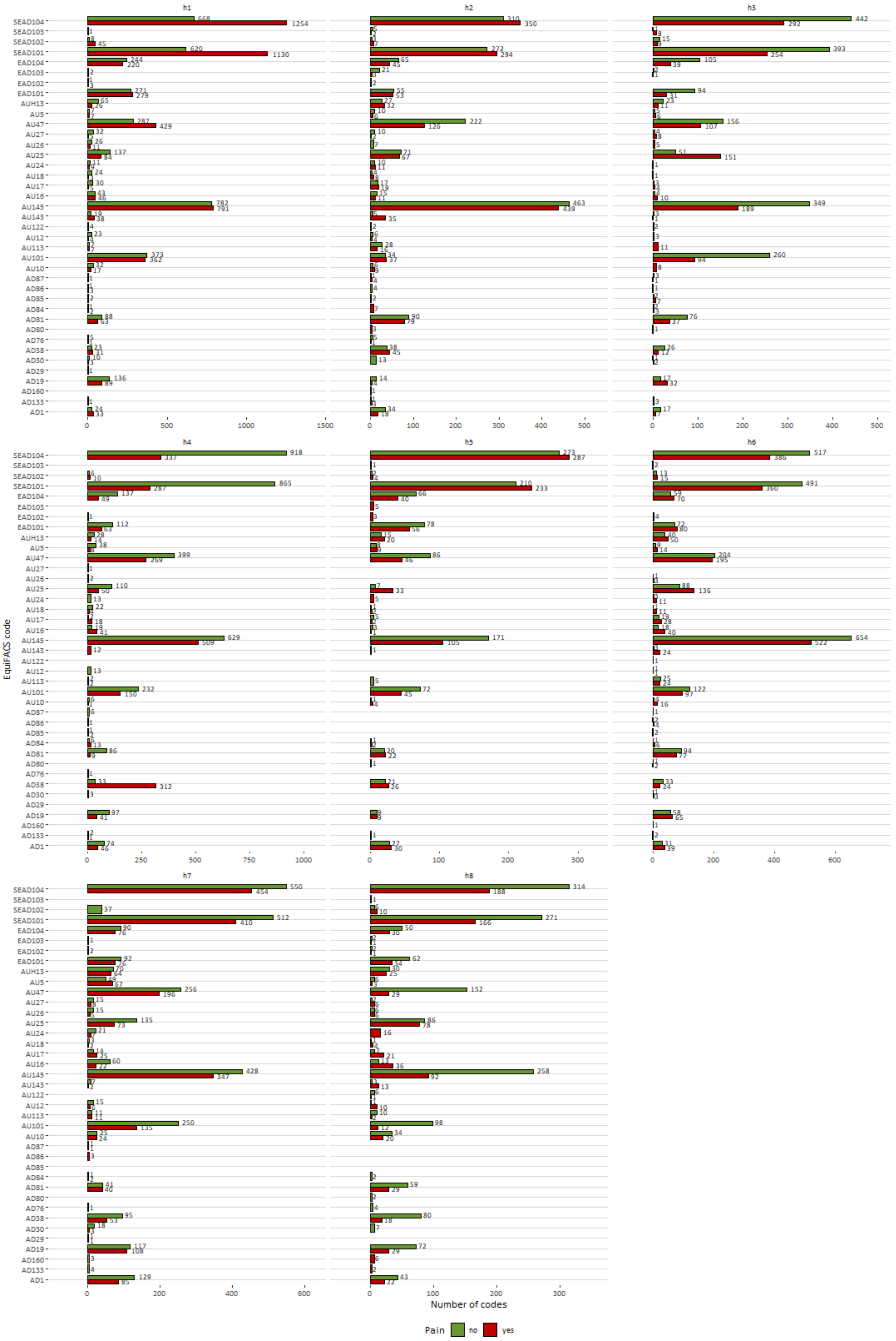

Supplement: Supplementary file 2 — Supplementary Information 2. [file 41598_2023_50383_MOESM2_ESM.pdf]
